# The Practice of Medicine in Oregon

**Published:** 1869-11

**Authors:** 


The Practice of Medicine in Oregon.—A subscriber in Oregon writes as follows: In some respects this is a good country to practice medicine in. The prices are almost double those charged East, payable in coin, but no attention is paid to medical ethics; each one does what he considers right in his own eyes. There is a Medical School at Salem —the capital. The number in attendance is small, and the graduates few, and not very well qualified. The physicians and surgeons of the regular school, in the cities of Portland and Salem, are well qualified, and will compare favorably with those of any eastern city. They are generally graduates of English, New York and Philadelphia schools.

